# Kinetic Prediction of Fast Curing Polyurethane Resins by Model-Free Isoconversional Methods

**DOI:** 10.3390/polym10070698

**Published:** 2018-06-23

**Authors:** Michael Stanko, Markus Stommel

**Affiliations:** Chair of Plastics Technology, TU Dortmund University, Leonhard-Euler-Str. 5, D-44227 Dortmund, Germany; markus.stommel@tu-dortmund.de

**Keywords:** isoconversional methods, reaction kinetics, fast curing polyurethane resin, differential scanning calorimetry

## Abstract

In this work, the characterisation of reaction kinetics of a methylene diphenyl diisocyanate (MDI)-based fast curing polyurethane resin (PUR) and the mathematical description of its curing process are presented. For the modelling of the reaction process isoconversional methods, which are also called model-free approaches, are used instead of model-based approaches. One of the main challenges is the characterisation of a reactive system with a short pot life, which already starts to crosslink below room temperature. The main focus is the evaluation of the applicability of isoconversional methods for predicting the reaction kinetics of fast curing polyurethane resins. In order to realise this, a repeatable methodology for the determination of time- and temperature-dependent reaction curves using differential scanning calorimetry (DSC) is defined. The cure models defined by this method serve as the basis for process simulations of PUR processing technologies such as resin transfer moulding (RTM) or reactive injection moulding (RIM) and reactive extrusion (REX). The characterisation of the reaction kinetics using DSC measurements is carried out under isothermal and non-isothermal conditions. Within this work isoconversional methods have been applied successfully to experimentally determined DSC data sets. It is shown that the reaction kinetics of fast curing polyurethane resins can be predicted using this methods. Furthermore, it is demonstrated that the time-dependent change of conversion of the considered polyurethane under isothermal curing conditions can also be predicted using isoconversional methods based on non-isothermal DSC measurements. This results in a significant reduction in the experimental effort required to characterise and model the curing process of polyurethanes.

## 1. Introduction

The processing of thermoset moulding materials like polyurethanes (PUR) is a very important field of plastics technology. The various properties of this material, especially the wide range of mechanical properties, are apparent in various application areas like the automotive industry, the construction industry as well as in products for everyday domestic needs. The mechanical properties of polyurethanes, depending on their chemical structure, provide the entire spectrum of material behaviour of engineering plastics, from rubber-elastic to hard elastic brittle deformation behaviour.

Therefore, it can be assumed that due to the application-specific variable material properties the importance of polyurethanes in all areas of technical applications will increase. In order to meet the requirements of modern development processes, it is necessary to carry out specific research and further developments, especially in the area of numerical simulation of polyurethane manufacturing processes, the calculation of mechanical stresses in polyurethane components and the prediction of process specific component properties.

Numerical process and structure simulations are now an essential part of the development processes of plastic components due to the complexity of the material behaviour of plastics. For polyurethane processing, the already published studies on process simulation can be divided into the manufacturing processes reactive injection moulding (RIM) [1,2], resin transfer moulding (RTM) [3] and reactive extrusion (REX) [4]. Within these categories, it is to be distinguished whether the simulations are used to illustrate the manufacturing process of a product made of a compact or foamed PUR material. For example, [5,6,7,8,9,10,11] shows approaches for the numerical simulation of the RIM manufacturing process for polyurethane foams. In addition, some authors such as Riviere et al. [12] take up special methods of plastics processing such as the rotational moulding process for reactive moulding resins.

All process simulations have in common that the characterisation and modelling of the reaction kinetics of the curing process is required for their definition. Regarding the characterisation and modelling of the reaction kinetics, studies on pure PUR systems as well as composite materials [13,14] can be found in the literature. The authors also frequently consider the chemo-rheological behaviour, which is related to the conversion during the curing process and leads to an increase in viscosity. Examples are the works of Chiacchiarelli et al. [15], Dimier et al. [16] or Sun et al. [17] which illustrates the characterisation of the curing process of polyurethane systems using isothermal and non-isothermal differential scanning calorimetry (DSC) measurements as well as the representation of the experimentally determined data by corresponding cure models.

In [18], Fernandez d’Arlas et al. applies so-called isoconversional methods to describe the curing process of a HDI-based polyurethane under isothermal conditions. The differential method by Friedman [19] and the approaches of Flynn-Wall-Ozawa [20] and Kissinger-Akahira-Sunose [21] are used and compared to the model-based autocatalytic approach by Kamal-Sourour [22]. In the context of this work, the investigations of Fernandez d’Arlas et al. [18] are extended by the description of non-isothermal curing processes using a method described in [23] by Vyazovkin and applied to an MDI-based polyurethane.

The model accuracy is strongly dependent on the quality of the experimentally determined reaction processes. It is important to ensure that the exothermic curing reactions of the considered resins are detected precisely using thermal analysis methods. Difficult test conditions are especially present if fast curing resins are the subject of the reaction kinetics characterisation. In contrast to conventional resins, which are treated in many published papers, fast curing resins exhibit no significant inhibition time. In many cases, resins of this type already react well below room temperature. This results in particular difficulties during the thermal analysis of the reaction process and a potential negative influence on the quality of the measured data for modelling the curing kinetics. For this reason, a repeatable measurement methodology has to be developed for the characterisation of such resins and their suitability has to be evaluated within the modelling procedure.

## 2. Materials and Methods

### 2.1. Materials

In this work a commercially available polyurethane resin system of the type NEUKADUR MultiCast 7 made by Altropol Kunststoffe GmbH (Stockelsdorf, Germany) was used. The resin is an unfilled fast curing resin. The PUR-System consists of a polyol formulation of branched polyesters and polyethers and 4,4′-methylene diphenyl diisocyanate (4,4′-MDI). The polyol and isocyanate have to be mixed in a ratio of 100:80 (parts by weight). For the thermal characterisation, a batch of 40 g was prepared for each measurement. From this batch, a sample was taken for the thermal characterisation using a micropipette and placed in a DSC pan.

### 2.2. Differential Scanning Calorimetry

**Figure 1:**
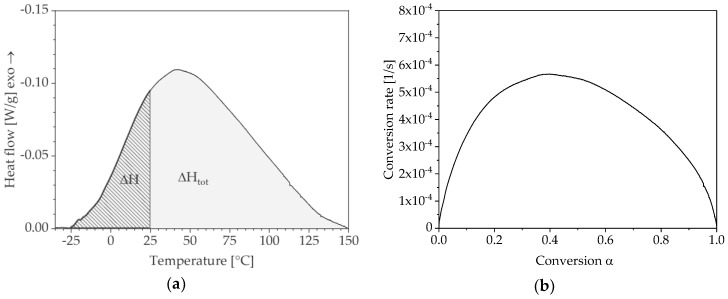
Determination of reaction-specific parameters based on differential scanning calorimetry (DSC) thermograms: (**a**) reaction enthalpy; (**b**) conversion rate against conversion.

The characterisation of reactive moulding materials such as polyurethane as well as epoxy or polyester resins is usually carried out by means of differential scanning calorimetry (DSC). The exothermic polymerisation reaction is reflected in the thermograms of a DSC measurement curve so that its time and temperature-dependent progression as well as the reaction enthalpy ΔH (Figure 1) and conversion α can be determined:

(1) $$\alpha =\frac{\Delta H}{\Delta H_{tot}}$$

For isothermal measurements, the total enthalpy $\Delta H_{tot}$ results from the sum of the enthalpy reacted during isothermal curing $\Delta H_{iso}$ and the residual enthalpy $\Delta H_{res}$ which is determined by a subsequent non-isothermal DSC measurement.

Based on these, specific characteristics of the reaction can be derived such as the conversion rate dα/dt depending on conversion α (Figure 1) which are used for the definition of appropriate cure models.

In this study, a differential scanning calorimeter of the type DSC8500 with an intracooler from PerkinElmer (Waltham, MA, USA) was used. Curing processes under both isothermal and non-isothermal conditions were considered during characterisation. The characterisation was performed for non-isothermal measurements in the temperature range of −50–150 °C. Heating rates of 3, 4, 5 and 7.5 °C/min were considered. In isothermal DSC measurements, curing temperatures in the range of 30–80 °C with a step of 10 °C were investigated. The curing temperature was applied using a heating rate of 700 °C/min to take into account as much of the heat of reaction as possible during the characterisation and subsequent modelling. After the sample preparation, the DSC pans were placed in the measurement cell, which had already been pre-cooled to a temperature of −50 °C. After a short isothermal holding phase for temperature homogenisation, the measurements were initiated. Due to the fact that the resin system starts to react at temperatures well below room temperature, the isothermal DSC measurements were also started at a temperature of −50 °C, unlike to other works where the cell is pre-tempered to the corresponding curing temperature. The sample weights lie in the range between 6–10 mg.

### 2.3. Reaction Kinetic Modelling

The conversion rate dα/dt of thermal activated processes can be described by the following equation:

(2) $$\frac{d\alpha }{dt}=k\left(T\right)f\left(\alpha \right),$$

where k(T) is the temperature-dependent rate constant and f(α) is the reaction model.

In this case, k(T) can be described by the Arrhenius equation:

(3) $$k\left(T\right)=A\;exp\left(-\frac{E}{RT}\right),$$

where A is the pre-exponential factor, E is the activation energy and R is the gas constant.

Different approaches for describing the reaction processes of thermoset materials can be found in the literature. Most of them can be divided into model-fitting and model-free approaches [24,25]. The latter are also called isoconversional methods. For the description of the reaction kinetics using the model fitting approach, a reaction model f(α) which is a conversion-dependent function is used. Some of these kinetic models are listed in Table 1. Other kinetic models can be found in the literature, such as in [26,27,28].

Many reactions as well as the curing processes of thermosets show a complex and multi-step behaviour. Often different reaction mechanisms can be observed due to parallel reactions and their mutual influence. Therefore, a constant activation energy can no longer be assumed in reactions like this. A mathematical description using established reaction models as illustrated by some examples in Table 1 and the determination of the activation energy using an Arrhenius plot is therefore in many cases only partially possible or too inaccurate. 

Isoconversional or model-free methods are based on the isoconversional principle, which eliminates the requirement of using an already existing reaction model or deriving a new model. In addition, these methods take into account the dependence of the activation energy E on the reaction conversion α. The isoconversional principle can be derived from the logarithmic derivation of Equation (2):

(4) $${\left[\frac{\partial \ln d\alpha /dt}{\partial T^{-1}}\right]}_{\alpha }={\left[\frac{\partial \ln k\left(T\right)}{\partial T^{-1}}\right]}_{\alpha }+{\left[\frac{\partial \ln f\left(\alpha \right)}{\partial T^{-1}}\right]}_{\alpha }.$$

For α=const also f(α) becomes constant and Equation (3) is reduced to the following relationship:

(5) $${\left[\frac{\partial \ln d\alpha /dt}{\partial T^{-1}}\right]}_{\alpha }=-\frac{E_{\alpha }}{R}.$$

The isoconversional principle indicates that the rate of conversion of a reaction for a defined conversion is only a function of temperature. The conversion-dependent activation energy $E_{\alpha }$ is determined using the temperature dependence of the conversion rates for a constant value of the conversion (isoconversional state), which has been obtained for different heating rates of non-isothermal DSC measurements. As shown in Equation (5), the knowledge of an appropriate reaction model f(α) is not required.

Isoconversional methods are subdivided into differential and integral methods. The differential method according to Friedman [19] can be derived from Equation (2) and is defined by the following relationship:

(6) $$\ln{\left(\frac{d\alpha }{dt}\right)}_{\alpha ,i}=\ln\left[f\left(\alpha \right)A_{\alpha }\right]-\frac{E_{\alpha }}{RT_{\alpha ,i}},$$

where i is the index for different temperature programs of the DSC measurement. From the slope of a plot of ln(dα/dt) in relation to the reciprocal temperature 1/$T_{\alpha ,i}$ the corresponding values of the activation energy $E_{\alpha }$ are determined for defined conversions α. As shown in [29] Equation (6) can be applied to simulate isothermal as well as non-isothermal curing processes.

Besides differential methods, there are also integral approaches. Based on Equation (2) and using the integral form of the reaction model g(α):

(7) $$g\left(\alpha \right)=\;\overset{\alpha }{\underset{0}{\int }}\frac{d\alpha }{f\left(\alpha \right)},\;$$

the following relationship can be derived:

(8) $$g\left(\alpha \right)=\overset{t}{\underset{0}{\int }}exp\left(-\frac{E}{RT}\right)dt.$$

For non-isothermal applications under constant temperature changes, this relationship is defined as follows:

(9) $$g\left(\alpha \right)=\frac{A}{\beta }\overset{T}{\underset{T_{0}}{\int }}exp\left(-\frac{E}{RT}\right)dT=\frac{A}{\beta }I\left(E,T\right),$$

where β is the heating rate of the temperature program. Due to the fact that the resulting temperature integral I(E,T) has no analytical solution, approximations or numerical integration methods are used to solve Equation (9). One of these approximations is proposed by Doyle [30] and used in the isoconversional methods of Flynn and Wall [31] and Ozawa [20]:

(10) $$\ln\left(\beta \right)=Const.-\frac{1.05E_{\alpha }}{RT_{\alpha ,i}}.$$

Further approximations are, for example, the Kissinger-Akahira-Sunose equation [21]:

(11) $$\ln\left(\frac{\beta }{T_{\alpha ,i}^{2}}\right)=Const.-\frac{E_{\alpha }}{RT_{\alpha ,i}},$$

or the approximation of Starink [32]:

(12) $$\ln\left(\frac{\beta }{T_{\alpha ,i}^{1,92}}\right)=Const.-\frac{E_{\alpha }}{RT_{\alpha ,i}}.$$

The conversion-dependent activation energy for chosen conversions α is determined according to the approximation method by calculating the slope of the plots of ln(β), ln(β/$T_{\alpha ,i}^{2})$ or ln(β/$T_{\alpha ,i}^{1,92})$ against the reciprocal temperature 1/$T_{\alpha ,i}$.

Another method is described in [23] by Vyazovkin. The determination of the activation energy for a certain value of the conversion is carried out by solving the temperature integral $I\left(E_{\alpha },T_{\alpha ,i}\right)$ using numerical integration and minimization of the following equation:

(13) $$\Phi (E_{\alpha })=\overset{n}{\underset{i=1}{\sum }}\overset{n}{\underset{j\neq i}{\sum }}\frac{I\left(E_{\alpha },T_{\alpha ,i}\right)\beta_{j}}{I\left(E_{\alpha },T_{\alpha ,j}\right)\beta_{i}}.$$

With isoconversional methods, not only the conversion-dependent activation energy but also the pre-exponential factor A, which also depends on the conversion and a generic reaction model f(α) can be determined. However, these are not required for the subsequent prediction of curing kinetics but may help in the interpretation of the observed reaction mechanisms.

In this work, the so-called compensation effect is used to determine the pre-exponential factor. A second method for determining the reaction model and the pre-exponential factor using so-called *y*(α) or *z*(α) master plots is not considered in this work. Contributions in which this approach is treated can be found in [27,33,34]. In addition, composite approaches are presented in [35,36]. 

**Figure 2:**
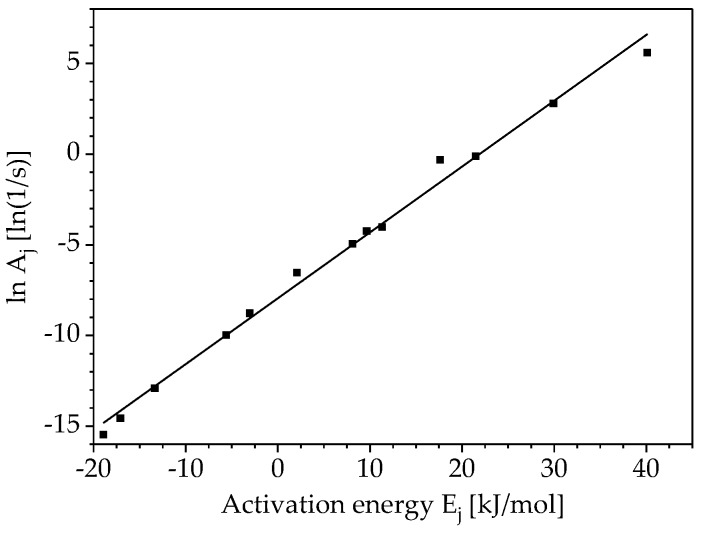
Exemplary illustration of a compensation line of a polyurethane resin (PUR) for a defined heating rate β = 3 °C/min applying the models 1–13 from Table 1.

The compensation effect takes up the fact that experimentally determined reaction kinetics can be represented with more than one kinetic triplet consisting of the reaction model f(α), the activation energy E and the pre-exponential factor A. This is caused by the simultaneous change of temperature T and conversion α during non-isothermal reaction processes and the fact that the reaction model f(α) and the rate constant k(T) cannot be clearly separated from each other. Using a plot of the conversion rate dα/dt versus temperature T at a selected heating rate β and applying a model fitting method using Equation (14):

(14) $$\ln\left(\frac{1}{f_{j}\left(\alpha \right)}\frac{d\alpha }{dt}\right)=\ln A_{j}-\frac{E_{j}}{RT},$$

compensation lines are determined. For this purpose, the dα/dt-plot is fitted with the reaction models $f_{j}\left(\alpha \right)$, given in Table 1. The result of each fit as a pair of values for $\ln A_{j}$ and $E_{j}$ are plotted like in Figure 2 and approximated by the following equation, describing the compensation effect:

(15) $$\ln A_{j}=aE_{j}+b.$$

Using the obtained factors a and b of the compensation effect and the previously calculated conversion-dependent activation energy $E_{\alpha }$, the pre-exponential factor $A_{\alpha }$ can be determined as a function of conversion:

(16) $$\ln A_{\alpha }=aE_{\alpha }+b.$$

With $E_{\alpha }$ and $A_{\alpha }$ a reaction model in differential form:

(17) $$f\left(\alpha \right)=\beta {\left(\frac{d\alpha }{dT}\right)}_{\alpha }{\left[A_{\alpha }exp\left(-\frac{E_{\alpha }}{RT_{\alpha }}\right)\right]}^{-1},$$

or integral form can be derived:

(18) $$g\left(\alpha \right)=\frac{A_{\alpha }}{\beta }\overset{T_{\alpha }}{\underset{0}{\int }}exp\left(-\frac{E_{\alpha }}{RT}\right)dT.$$

For the subsequent application of the model in the framework of process simulations, the possibility of predicting reaction processes for other curing conditions than those considered in the characterisation is necessary. Using isoconversional methods, both isothermal and non-isothermal reaction curves can be predicted. Isothermal curing processes can be determined by means of a single non-isothermal reaction at a defined heating rate β using the following equation.

(19) $$t_{\alpha }=\overset{k}{\underset{i=1}{\sum }}t_{a,i}=\overset{k}{\underset{i=1}{\sum }}\frac{\int_{T_{\alpha ,i-1}}^{T_{\alpha }}exp\left(-\frac{E_{\alpha }}{RT}\right)dT}{\beta exp\left(-\frac{E_{\alpha ,i}}{RT_{0}}\right)}.$$

The time $t_{\alpha }$ until the conversion α is reached is calculated by summing the sectional integration over time.

Alternatively, a simplified approach can be used to model isothermal curing processes as shown in [37,38]. Based on Equation (8) the curing kinetic is predicted by numerical integration.

Using Equation (19) and the previously determined conversion-dependent activation energy $E_{\alpha }$, isothermal curing processes for different temperatures $T_{0}$ can be calculated. Equation (20) is used to predict non-isothermal reaction curves:

(20) $$\int_{t_{\alpha ,i-1}}^{t_{\alpha }}exp\left(-\frac{E_{\alpha }}{RT_{0}\left(t\right)}\right)dt=\int_{t_{\alpha ,i-1}^{*}}^{t_{\alpha }^{*}}exp\left(-\frac{E_{\alpha }}{RT\left(t\right)}\right)dt.$$

To predict the reaction process α-$t_{\alpha }$ under the influence of a temperature profile $T_{0}\left(t\right)$ Equation (20) has to be solved using a reference curve α-$t_{\alpha }^{*}$ with the temperature profile T(t).

When using isoconversional methods to describe the curing process of thermosets and to determine kinetic triplets, it should be considered that these have to be applied consistently, since the calculated parameters of integral and differential methods are not exchangeable. Although in [29] it is shown that Friedman’s approach and Vyazovkin’s modified method lead to comparable results.

For more detailed information on isoconversional methods of thermally activated processes, reference is made to Vyazovkin [24]. Vyazovkin’s work is the basis of the theoretical overview of isoconversional methods given above.

## 3. Results and Discussion

**Figure 3:**
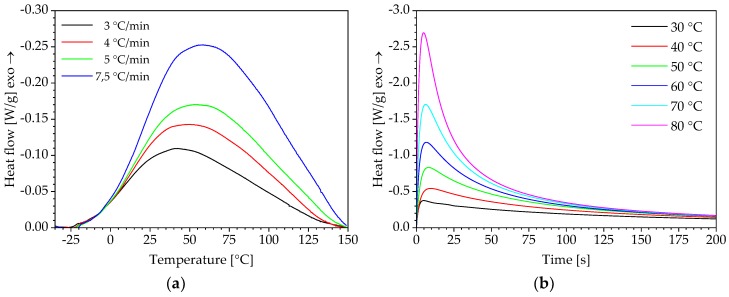
Thermograms of the curing reaction under: (**a**) non-isothermal and (**b**) isothermal conditions.

The results of the DSC measurements under isothermal and non-isothermal curing conditions are shown in Figure 3. The curves of the heat flow are obtained for four different heating rates (non-isothermal measurement) and six different curing temperatures (isothermal measurement) in relation to temperature and time.

The results indicate that the PUR system already starts to react at approximately −25 °C. For the thermal characterisation, this results in several challenges during preparation and handling of the sample. If possible, the curing reaction should be stopped until the measurement is started by pre-cooling the DSC measuring cell. However, the raw materials must also be mixed within an ice water bath or by using alternative cooling methods, as already described in some other studies. In any case, the individual measurements should always be carried out in the same way. The procedure described above for the preparation of the DSC samples and the calorimeter enables to determine thermograms that are suitable for the modelling of the reaction kinetics described below.

During the interpretation of curing processes, the effect of vitrification must be taken into account. With increasing degree of crosslinking while curing, the glass transition temperature increases. If the glass transition temperature reaches the level of the curing temperature, this leads to a rapid reduction in molecular chain mobility and, as a result, a decrease of the curing rate. The reaction becomes diffusion-controlled. Within a DSC thermogram this effect is expressed as a decreasing heat flow, which can be misinterpreted as a slowing down of the curing reaction. Therefore, vitrification occurs when the temperature of an isothermal curing process is below the glass transition temperature of a fully cured resin system $T_{g\infty }$. The glass transition temperature $T_{g\infty }$ of the PUR system discussed in this study is 58.3 °C. This means that when the polyurethane is cured, below this temperature, vitrification occurs.

In Table 2, the enthalpies of isothermal and non-isothermal calorimetric analyses are summarised. As shown in Table 2, the total enthalpy $\Delta H_{tot}$ of isothermal DSC measurements increases with increasing curing temperature. The reason for this is that conventional DSC measurements were carried out for the calorimetric investigations of this work. This results in an overlap of the glass transition (endothermic process) with the exothermic curing in the second non-isothermal DSC scan for measurements up to a curing temperature of $T_{cure}$ = 70 °C. So that the determination of residual enthalpy is affected by an error. Techniques such as temperature-modulated DSC measurement or the StepScan technology [39] can be used to observe the overlaid effects independently.

For the further considerations of this work, the average total enthalpy $\Delta H_{tot}$ = 166.6 J/g from the measurements with $T_{cure}\;$= 80–110 °C is used. 

**Figure 4:**
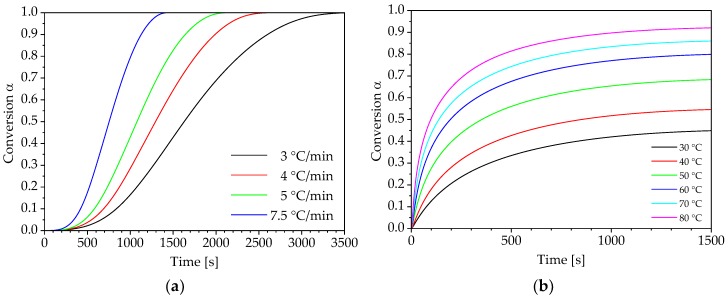
Progress of conversion: (**a**) non-isothermal curing and (**b**) isothermal conditions curing.

As shown in Figure 1a, the conversion can be determined from the thermograms. The resulting plots are shown in Figure 4 for both curing conditions and the corresponding variation levels.

Furthermore, the reaction processes determined under isothermal curing conditions are not considered at this point, because non-isothermal measurements are sufficient using isoconversional methods to define a complete model and predict both isothermal and non-isothermal reaction processes.

### 3.1. Determination of Conversion-Dependent Activation Energy

**Figure 5:**
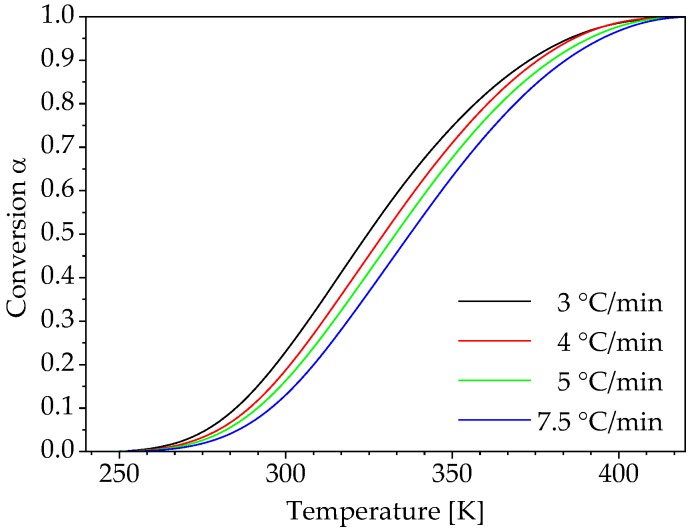
Temperature-dependent conversion under non-isothermal curing conditions.

The conversion rate dα/dt is determined from the conversion α over time t (Figure 4). The conversion rate is necessary for the determination of the activation energy $E_{\alpha }$ if the differential isoconversional method according to Friedman is used. However, in integral isoconversional methods, the dependency of conversion α on temperature T or reciprocal temperature 1/T are used to calculate the activation energy. Figure 5 shows the change in conversion over the temperature for the considered heating rates. 

**Figure 6:**
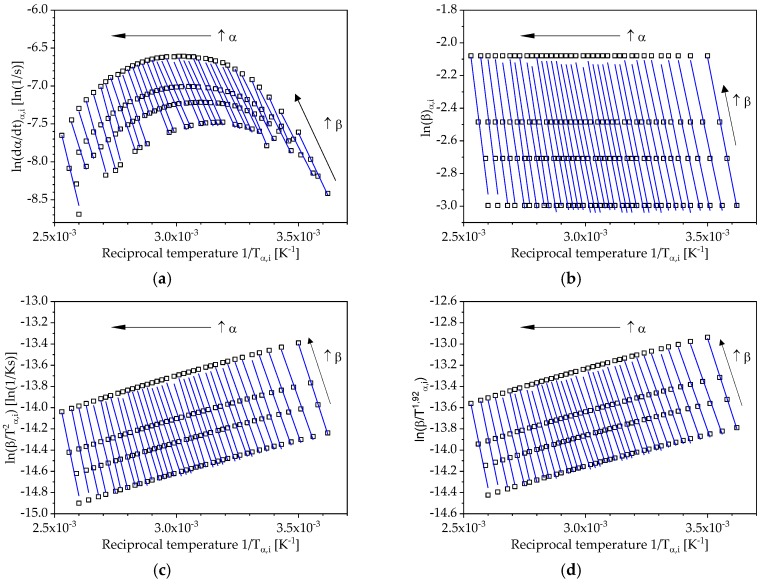
Isoconversional plots according to: (**a**) Friedman; (**b**) Flynn-Wall-Ozawa; (**c**) Kissinger-Akahira-Sunose; (**d**) Starink.

According to the isoconversional principle, the conversion-dependent activation energy $E_{\alpha }$ is determined using the temperature dependence of the curing process for different heating rates. The corresponding reciprocal temperatures are ascertained for defined values of conversion α for the heating rates 3, 4, 5 and 7.5 °C/min and displayed according to Equation (6) and Equations (10)–(12) in appropriate plots as illustrated in Figure 6. Within the framework of this work the evaluation and modelling of the reaction kinetics was carried out in the range of 0.05 to 0.95 with an interval of 0.025. The straight lines shown in Figure 6 represent these conversion values and are based on four points, which represent the applied heating rates. The differential isoconversional method according to Friedman and the integral approaches according to Flynn-Wall-Ozawa, Kissinger-Akahira-Sunose and Starink are considered here.

**Figure 7:**
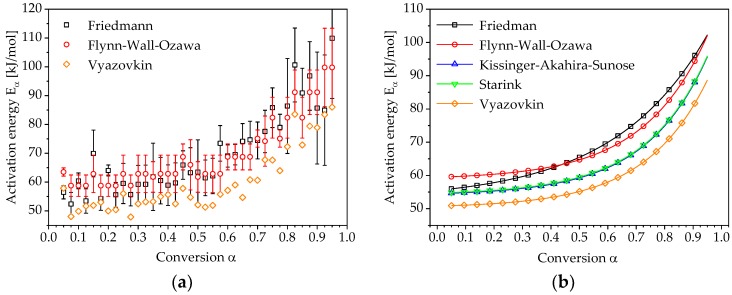
Dependence of activation energy in relation to reaction conversion: (**a**) experimental data; (**b**) data fitted to Equation (21).

The corresponding conversion-dependent activation energy can be determined from the slopes of these lines. Figure 7a shows the activation energies obtained using a differential (Friedman) and integral (Flynn-Wall-Ozawa) isoconversional method. In addition, the plot shows the course of the activation energy, calculated according to Vyazovkin using numerical integration and an algorithm for minimization of Equation (13). The resulting plots can be approximated by the following exponential function:

(21) $$E_{\alpha }=E_{0}+C\;exp\left(-\frac{\alpha }{b}\right),$$

where $E_{0}$, C and b are fitting parameters. The approximated curves of the activation energies for all evaluated methods are shown in Figure 7b.

It can be seen that the curves of the integral isoconversional methods and the activation energy determined by Vyazovkin’s method are almost identical and differ only in their level. The activation energy curve determined by Friedman shows a higher slope at the beginning of the reaction process, which becomes closer to that of the integral methods as it progresses further.

### 3.2. Determination of Pre-Exponential Factor and Reaction Model

**Figure 8:**
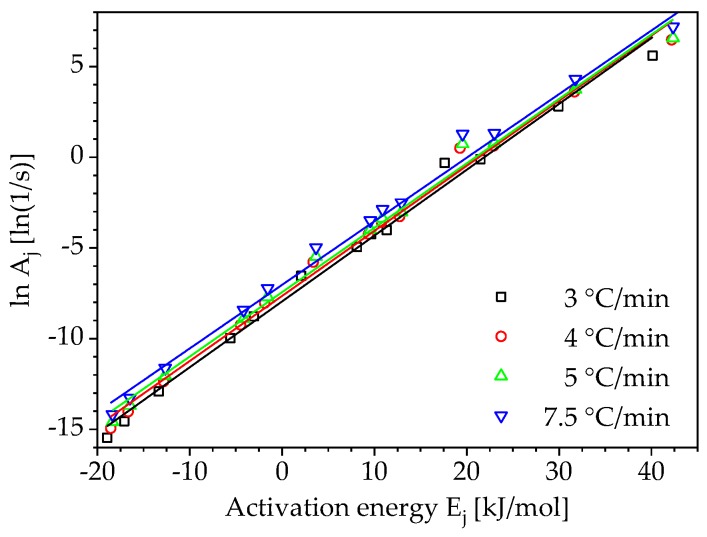
Compensation lines for different heating rates (☐ calculated $\ln A_{\alpha }-E_{\alpha }$ pair, — linear fit).

Using isoconversional methods also the pre-exponential factor and the reaction model of a curing reaction can be determined, even though they are not necessary for the prediction of the reaction kinetics for states outside the conditions considered in the experiment. For the computation of the pre-exponential factor and the reaction model only the calculated activation energy according to Vyazovkin is considered in the following. Applying the kinetic models 1–13 from Table 1, the already mentioned above compensation lines are determined according to Equation (14). Using the thirteen kinetic models from Table 1, thirteen pairs of activation energy and pre-exponential factor values are determined for each heating rate as shown in Figure 8.

**Figure 9:**
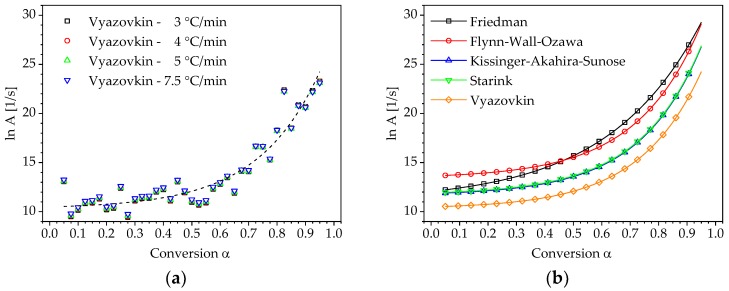
Correlation between $\ln A_{\alpha }$ and the conversion α : (**a**) calculated using $E_{\alpha }$ determined by Vyazovkin’s method; (**b**) calculated using $E_{\alpha }$ determined by different isoconversional methods for the heating rate β = 3 °C/min.

The compensation effect factors a and b (Equation (15)) can be defined from the logarithmic plot of the pre-exponential factor as a function of the activation energy. The compensation effect factors as well as the activation energy are then used to obtain a conversion-dependent pre-exponential factor according to Equation (16). The curves of the pre-exponential factor for different heating rates using the activation energy determined by Vyazovkin’s method are shown in Figure 9a. For completeness, Figure 9b illustrates the calculated curves of the pre-exponential factors using the other activation energies for the heating rate β= 3 °C/min.

**Figure 10:**
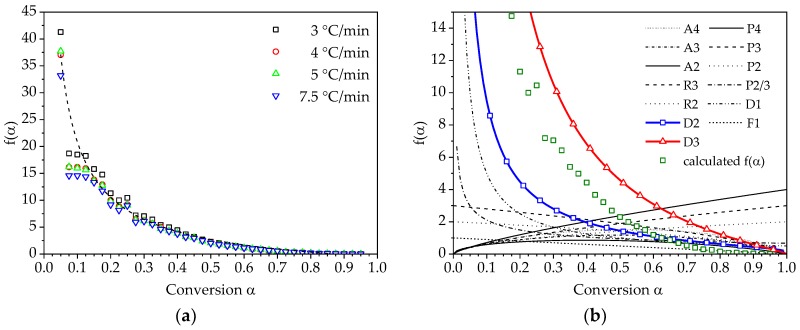
Numerical reconstruction of the reaction model in integral form f(α): (**a**) calculated reaction model using the α−T− dependencies for different heating rates; (**b**) comparison of the calculated model with theoretical models from Table 1

The calculation of a generic reaction model f(α) and the comparison with theoretical heterogeneous kinetic models as listed in Table 1 can be used in some cases as an additional information for understanding the reaction mechanism of the observed curing process. Figure 10a illustrates the curves of the generic reaction model in integral form for different heating rates resulting from the solution of Equation (17). A comparison between the numerically determined reaction model and chosen theoretical models from Table 1 can be seen in Figure 10b.

It can be seen that the reaction model of the considered PUR system has a characteristic that lies between that of the D2 and D3 model. These models represent the two- and three-dimensional mathematical description of chemical reactions coupled with diffusion processes. Therefore, for the modelling of curing kinetic using a model-based approach, the D3 model would be used, as this kinetic model represents the investigated curing reaction most closely.

### 3.3. Prediction of Reaction Kinetics

**Figure 11:**
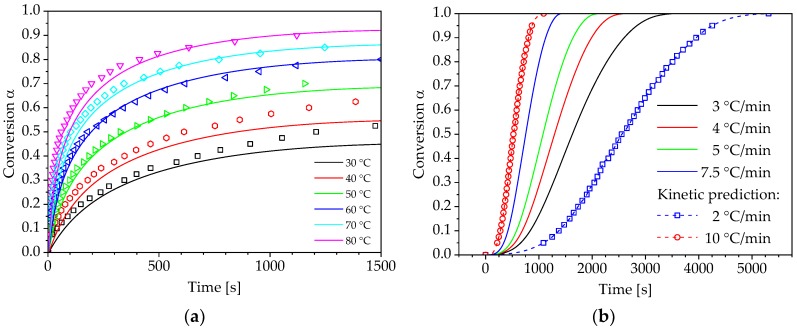
Kinetic prediction of the PUR curing process (— experimental data, ☐ model prediction): (**a**) isothermal curing conditions; (**b**) non-isothermal curing conditions.

In Section 3.2 the determination of the pre-exponential factor as well as the calculation of a reaction model and its comparison to selected theoretical kinetic models were presented, which is the fundament for a model-based description of the PUR curing process. However, the main advantage of isoconversional methods is the possibility of predicting the reaction kinetics without the knowledge of a particular reaction model and the pre-exponential factor. After determining the activation energy as shown in Section 3.1., Equations (19) and (20) can be used to predict the progression of isothermal and non-isothermal curing processes. For this purpose, only a non-isothermal curve of conversion over time and the activation energy $E_{\alpha }$ are required. As a result, the computation effort can be reduced by the operations of the previous section. The results of the curing kinetics predictions for isothermal and non-isothermal curing conditions are shown in Figure 11a,b.

The modelling of isothermal curing exhibits a very good agreement with the experimentally determined progressions of the reaction conversion. However, the model does not accurately depict the courses of conversion for curing conditions $T_{cure}<T_{g\infty }$, because it does not take into account the vitrification below the glass transition temperature $T_{g\infty }$. In addition, isoconversional methods can be used to determine non-isothermal curing processes which are outside the range investigated in the experiments. This is exemplarily shown in Figure 11b for the heating rates β= 2 °C/min and β= 10 °C/min.

## 4. Conclusions and Outlook

In the scope of this work, the characterisation of the reaction kinetics of fast curing MDI-based PUR resins using DSC was presented. The investigated PUR system has no significant inhibition time and reacts already well below room temperature. In this context, a repeatable methodology for determining the reaction process for isothermal and non-isothermal curing conditions was prepared and successfully applied. The experimental data obtained were used for the mathematical description and modelling of curing processes using isoconversional methods. It could be shown that the crosslinking reaction of fast curing PUR resins can also be modelled with high accuracy using this method for curing conditions $T_{cure}>T_{g\infty }$. Below the glass transition temperature $T_{g\infty }$, a deviation is observed between the experimentally determined data and the model prediction, which is caused by vitrification. The results of this work are the basis for the implementation of curing processes in numerical process simulations of PUR manufacturing methods such as RIM, RTM or REX. Furthermore, it could be shown that DSC measurements under isothermal curing conditions can be omitted in any future investigations. The resulting reaction characteristics can be reconstructed very accurately using a curing model based solely on non-isothermal DSC measurements. This leads to a significant reduction in the time needed for the thermal characterisation of curing PUR resins.

## Figures and Tables

**Table 1:** Chosen kinetic models to describe thermally activated processes [24].

	Code	Reaction model	f(α)	g(α)
1	P4	Power law	$4\alpha^{3/4}$	$\alpha^{1/4}$
2	P3	Power law	$3\alpha^{2/3}$	$\alpha^{1/3}$
3	P2	Power law	$2\alpha^{1/2}$	$\alpha^{1/2}$
4	P2/3	Power law	$2/3\alpha^{-1/2}$	$\alpha^{3/2}$
5	D1	One-dimensional diffusion	$1/2\alpha^{-1}$	$\alpha^{2}$
6	F1	Mampel (first-order)	1−α	−ln(1−α)
7	A4	Avrami-Erofeev	$4\left(1-\alpha \right){\left[-\ln\left(1-\alpha \right)\right]}^{3/4}$	${\left[-\ln\left(1-\alpha \right)\right]}^{1/4}$
8	A3	Avrami-Erofeev	$3\left(1-\alpha \right){\left[-\ln\left(1-\alpha \right)\right]}^{2/3}$	${\left[-\ln\left(1-\alpha \right)\right]}^{1/3}$
9	A2	Avrami-Erofeev	$2\left(1-\alpha \right){\left[-\ln\left(1-\alpha \right)\right]}^{1/2}$	${\left[-\ln\left(1-\alpha \right)\right]}^{1/2}$
10	D3	Three-dimensional diffusion	$2{\left(1-\alpha \right)}^{2/3}{\left[1-{\left(1-\alpha \right)}^{1/3}\right]}^{-1}$	${\left[1-{\left(1-\alpha \right)}^{1/3}\right]}^{2}$
11	R3	Contracting sphere	$3{\left(1-\alpha \right)}^{2/3}$	$1-{\left(1-\alpha \right)}^{1/3}$
12	R2	Contracting cylinder	$2{\left(1-\alpha \right)}^{1/2}$	$1-{\left(1-\alpha \right)}^{1/2}$
13	D2	Second-order	${\left(1-\alpha \right)}^{2}$	${\left(1-\alpha \right)}^{-1}-1$

**Table 2:** Kinetic parameters obtained during isothermal and non-isothermal DSC measurements.

DSC _isotherm_				DSC _non-isotherm_	
$T_{cure}$ [°C]	$\Delta H_{iso}$ [J/g]	$\Delta H_{res}$ [J/g]	$\Delta H_{tot}$ [J/g]	β [°C/min]	$\Delta H_{dyn}$ [J/g]
30	75.3	46.2	121.5	3	193.3
40	91.5	32.2	123.7	4	194.0
50	114.4	24.4	138.8	5	188.8
60	133.7	19.6	153.3	7.5	187.4
70	143.3	14.7	158.0		
80	155.4	12.9	168.3		
90	158.9	5.9	164.8		
100	161.5	1.5	163.0		
110	170.2	0	170.2		

## References

[1] Lee W.H., Lee S.W., Kang T.J., Chung K., Youn J.R. (2002). Processing of polyurethane/polystyrene hybrid foam and numerical simulation. Fibers Polym..

[2] Samkhaniani N., Gharehbaghi A., Ahmadi Z. (2013). Numerical simulation of reaction injection molding with polyurethane foam. J. Cell. Plast..

[3] Canal L.P., Benavente M., Hausmann M., Michaud V. (2015). Process-induced strains in RTM processing of polyurethane/carbon composites. Compos. Part A.

[4] Puaux J.-P., Cassagnau P., Bozga G., Nagy I. (2006). Modeling of polyurethane synthesis by reactive extrusion. Chem. Eng. Process. Process Intensif..

[5] Seo D., Ryoun Youn J., Tucker C.L. (2003). Numerical simulation of mold filling in foam reaction injection molding. Int. J. Numer. Methods Fluids.

[6] Seo D., Youn J.R. (2005). Numerical analysis on reaction injection molding of polyurethane foam by using a finite volume method. Polymer.

[7] Tröltzsch J., Schäfer K., Niedziela D., Ireka I., Steiner K., Kroll L. (2017). Simulation of RIM-process for Polyurethane Foam Expansion in Fiber Reinforced Sandwich Structures. Procedia CIRP.

[8] Geier S., Winkler C., Piesche M. (2009). Numerical Simulation of Mold Filling Processes with Polyurethane Foams. Chem. Eng. Technol..

[9] Abdessalam H., Abbès B., Guo Y.Q., Kwassi E., Romain J.L. (2013). Numerical Simulation of Polyurethane Foaming Process Using Finite Point Method. AMR.

[10] Abdessalam H., Abbès B., Li Y., Guo Y.-Q., Kwassi E., Romain J.-L., Saanouni K. (2016). Prediction of acoustic foam properties by numerical simulation of polyurethane foaming process. MATEC Web Conf..

[11] Bikard J., Bruchon J., Coupez T., Silva L. (2007). Numerical simulation of 3D polyurethane expansion during manufacturing process. Colloids Surf. A Physicochem. Eng. Asp..

[12] Riviere S., Khelladi S., Farzaneh S., Bakir F., Tcharkhtchi A. (2013). Simulation of polymer flow using smoothed particle hydrodynamics method. Polym. Eng. Sci..

[13] Chiacchiarelli L.M., Puri I., Puglia D., Kenny J.M., Torre L. (2012). Cure kinetics of a highly reactive silica–polyurethane nanocomposite. Thermochim. Acta.

[14] Kim D.S., Kim J.-T., Woo W.B. (2005). Reaction kinetics and characteristics of polyurethane/clay nanocomposites. J. Appl. Polym. Sci..

[15] Chiacchiarelli L.M., Kenny J.M., Torre L. (2013). Kinetic and chemorheological modeling of the vitrification effect of highly reactive poly(urethane-isocyanurate) thermosets. Thermochim. Acta.

[16] Dimier F., Sbirrazzuoli N., Vergnes B., Vincent M. (2004). Curing kinetics and chemorheological analysis of polyurethane formation. Polym. Eng. Sci..

[17] Sun X., Toth J., Lee L.J. (1997). Chemorheology of poly(urethane/isocyanurate) formation. Polym. Eng. Sci..

[18] Fernandez d’Arlas B., Rueda L., Stefani P.M., de la Caba K., Mondragon I., Eceiza A. (2007). Kinetic and thermodynamic studies of the formation of a polyurethane based on 1,6-hexamethylene diisocyanate and poly(carbonate-*co*-ester)diol. Thermochim. Acta.

[19] Friedman H.L. (1964). Kinetics of thermal degradation of char-forming plastics from thermogravimetry. Application to a phenolic plastic. J. Polym. Sci. C Polym. Symp..

[20] Ozawa T. (1965). A New Method of Analyzing Thermogravimetric Data. Bull. Chem. Soc. Jpn..

[21] Kissinger H.E. (1957). Reaction Kinetics in Differential Thermal Analysis. Anal. Chem..

[22] Kamal M.R., Sourour S. (1973). Kinetics and thermal characterization of thermoset cure. Polym. Eng. Sci..

[23] Vyazovkin S., Dollimore D. (1996). Linear and Nonlinear Procedures in Isoconversional Computations of the Activation Energy of Nonisothermal Reactions in Solids. J. Chem. Inf. Comput. Sci..

[24] Vyazovkin S. (2015). Isoconversional Kinetics of Thermally Stimulated Processes.

[25] Vyazovkin S., Wight C.A. (1999). Model-free and model-fitting approaches to kinetic analysis of isothermal and nonisothermal data. Thermochim. Acta.

[26] Ramis X., Cadenato A., Morancho J.M., Salla J.M. (2003). Curing of a thermosetting powder coating by means of DMTA, TMA and DSC. Polymer.

[27] Sbirrazzuoli N. (2013). Determination of pre-exponential factors and of the mathematical functions f(α) or G(α) that describe the reaction mechanism in a model-free way. Thermochim. Acta.

[28] Ramis X., Salla J.M., Cadenato A., Morancho J.M. (2003). Simulation of isothermal cure of A powder coating. J. Therm. Anal. Calorim..

[29] Fernandez X., Ramis X., Salla J.M. (2005). Cationic copolymerization of cycloaliphatic epoxy resin with an spirobislactone with lanthanum triflate as initiator. Thermochim. Acta.

[30] Doyle C.D. (1962). Estimating isothermal life from thermogravimetric data. J. Appl. Polym. Sci..

[31] Flynn J.H., Wall L.A. (1966). A quick, direct method for the determination of activation energy from thermogravimetric data. J. Polym. Sci. B Polym. Lett..

[32] Starink M.J. (2003). The determination of activation energy from linear heating rate experiments: A comparison of the accuracy of isoconversion methods. Thermochim. Acta.

[33] Gotor F.J., Criado J.M., Malek J., Koga N. (2000). Kinetic Analysis of Solid-State Reactions: The Universality of Master Plots for Analyzing Isothermal and Nonisothermal Experiments. J. Phys. Chem. A.

[34] Roşu D., Mititelu A., Caşcaval C.N. (2004). Cure kinetics of a liquid-crystalline epoxy resin studied by non-isothermal data. Polym. Test..

[35] Criado J.M., Pérez-Maqueda L.A., Gotor F.J., Málek J., Koga N. (2003). A unified theory for the kinetic analysis of solid state reactions under any thermal pathway. J. Therm. Anal. Calorim..

[36] Cadenato A., Morancho J.M., Fernández-Francos X., Salla J.M., Ramis X. (2007). Comparative kinetic study of the non-isothermal thermal curing of bis-GMA/TEGDMA systems. J. Therm. Anal. Calorim..

[37] Vyazovkin S. (1997). Evaluation of activation energy of thermally stimulated solid-state reactions under arbitrary variation of temperature. J. Comput. Chem..

[38] Adeli M., Zarnegar Z., Dadkhah A., Hossieni R., Salimi F., Kanani A. (2007). Linear-dendritic ABA triblock copolymers as nanocarriers. J. Appl. Polym. Sci..

[39] Vergnaud J.-W., Bouzon J., Vergnaud J.-W., Bouzon J. (1992). Differential Scanning Calorimetry. Cure of Thermosetting Resins.

